# Expression of Phosphomimetic OSTM1-T328E/S329D Variant Partially Restores Bone Resorption Defect in LRRK1-Deficient Mice †

**DOI:** 10.3390/biology15120964

**Published:** 2026-06-19

**Authors:** Anakha Udayakumar, Yian Chen, Haibo Zhao, Subburaman Mohan, Weirong Xing

**Affiliations:** 1 The Musculoskeletal Disease Center, Jerry L Pettis VA Medical Center, Loma Linda, CA 92357, USA; anakha.udayakumar@va.gov (A.U.); yian.chen2@va.gov (Y.C.); subburaman.mohan@va.gov (S.M.); 2 Graduate Schools, Loma Linda University, Loma Linda, CA 92354, USA; 3 Southern California Institute for Research and Education, Long Beach, CA 90815, USA; haibo.zhao@va.gov; 4 Department of Medicine, Loma Linda University, Loma Linda, CA 92354, USA

**Keywords:** LRRK1, OSTM1, osteoclast, bone resorption, knockout, gene knock-in, phosphorylation, bone, skeletal phenotype

## Abstract

LRRK1 is essential for osteoclast-mediated bone resorption, and loss of LRRK1 function causes osteopetrosis in mice and humans. However, the mechanisms by which LRRK1 regulates osteoclast activity remain incompletely defined. We previously identified that phosphorylation of OSTM1 at threonine 328 and serine 329 was compromised in LRRK1-deficient osteoclasts. To test the role for OSTM1 phosphorylation in LRRK1 regulation of osteoclast functions, we expressed a phosphomimetic OSTM1 variant in LRRK1-null osteoclasts. Overexpression of phosphomimetic, but not a dephosphomimetic variant, partially restored resorptive activity in LRRK1-deficient osteoclasts in vitro. To test OSTM1’s role in rescuing defective bone resorption in *Lrrk1-null* mice, we generated *Ostm1-T328E/S329D* knock-in (KI) mice and crossed them onto the *Lrrk1*-deficient background. *Ostm1-T328E/S329D* KI mice displayed normal skeletal development and bone remodeling. When crossed to the *Lrrk1*-deficient background, OSTM1-T328E/S329D expression increased osteoclast resorptive activity and bone formation and partially improved trabecular architecture, although bone volume remained unchanged. These findings demonstrate that OSTM1 phosphorylation contributes to LRRK1-dependent regulation of osteoclast function and identify the LRRK1–OSTM1 pathway as a mechanistic node controlling bone resorption. Our work provides new insight into the molecular basis of LRRK1-mediated osteoclast function and highlights OSTM1 phosphorylation as a potential therapeutic target for metabolic bone diseases.

## Simple Summary

LRRK1 is essential for osteoclast-mediated bone resorption, but its downstream effectors remain unclear. Since phosphorylation of OSTM1 was diminished in osteoclasts lacking LRRK1, we tested the role of OSTM1 phosphorylation in mediating LRRK1 effects in osteoclasts. Overexpression of a phosphomimetic OSTM1 variant (T328E/S329D) partially restored resorptive activity in LRRK1-deficient osteoclasts, whereas a dephosphomimetic form did not. *Ostm1-T328E/S329D* knock-in mice displayed normal skeletal development, but expression of this variant in *Lrrk1*-deficient mice enhanced osteoclast activity, increased bone formation, and partially improved resorption. These findings establish OSTM1 phosphorylation as an important component of the LRRK1 signaling axis and a potential target for modulating osteoclast function.

## 1. Introduction

Bone remodeling is a dynamic process driven by the coordinated actions of osteoblasts and osteoclasts to maintain skeletal integrity. Disruption of this balance underlies major metabolic bone diseases, including osteoporosis—marked by excessive bone resorption—and osteopetrosis, which results from impaired osteoclast function. With more than 10 million Americans affected by osteoporosis and tens of millions more at risk, there is a critical need for therapeutic strategies that prevent fragility fractures while preserving physiological bone remodeling [1]. Current antiresorptive agents effectively suppress osteoclastogenesis but can impair osteoclast-coupled bone formation when used long-term, highlighting the need for approaches that selectively modulate osteoclast function rather than broadly inhibiting differentiation [2,3,4].

Leucine-rich repeat kinase 1 (LRRK1) has emerged as an essential regulator of osteoclast resorptive activity [5,6]. *Lrrk1*-deficient mice develop severe osteopetrosis with markedly increased trabecular bone mass due to defective osteoclast function, despite largely intact osteoclast differentiation and normal bone formation [5]. Osteoclasts lacking LRRK1 are enlarged and TRAP-positive but fail to attach to bone surfaces or form functional sealing zones. Notably, *Lrrk1* knockout (KO) mice retain responsiveness to anabolic parathyroid hormone and are resistant to estrogen-deficiency-induced bone turnover, indicating that LRRK1 acts specifically within the osteoclast resorption machinery [5]. Consistent with these findings, individuals with biallelic *Lrrk1* mutations present with osteosclerotic metaphyseal dysplasia caused by dysfunctional osteoclasts [7,8,9]. These observations position LRRK1 as a promising therapeutic target for disorders of excessive bone resorption, yet the molecular mechanisms by which LRRK1 controls osteoclast function remain incompletely defined.

In our mechanistic studies, we previously demonstrated that LRRK1 regulates cytoskeletal organization, sealing zone assembly, and lysosomal exocytosis in osteoclasts. LRRK1-deficient osteoclasts fail to form ruffled borders, and LAMP2-positive lysosomes containing cathepsin K and vacuolar H^+^-ATPase are mislocalized and unable to secrete into the resorptive lacuna [5,10]. Proteomic analysis identified osteopetrosis-associated transmembrane protein 1 (OSTM1)—the β-subunit of the ClC-7 chloride/proton exchanger—as a potential downstream LRRK1 substrate [10]. OSTM1 was phosphorylated at threonine 328 and serine 329 in wild-type (WT) osteoclasts but not in LRRK1-deficient cells. The phosphorylation events are predicted to promote ClC-7/OSTM1 complex stability and lysosomal Cl^−^/H^+^ exchange. In other studies, it has been reported that loss-of-function mutations in either ClC-7 or OSTM1 caused severe osteopetrosis in humans and mice, and deletion of the OSTM1 C-terminal cytoplasmic domain (residues 266–338) produced a similar osteopetrosis phenotype [11,12,13,14,15]. In addition, OSTM1 is also known to interact with a motor protein of kinesin-5B, implicating it in lysosomal positioning and trafficking [16].

Given the essential role of OSTM1 in osteoclast function and our finding that OSTM1 phosphorylation is severely compromised in LRRK1-deficient osteoclasts, we hypothesized that LRRK1-mediated phosphorylation of OSTM1 at T328/S329 is essential for osteoclast resorptive activity. We further posited that restoring OSTM1 phosphorylation through expression of a phosphomimetic OSTM1 variant could rescue the osteopetrotic phenotype of *Lrrk1*-deficient mice. To directly test this mechanism in vivo, we generated knock-in (KI) mice in which endogenous *Ostm1* gene was replaced with a mutant allele to express phosphomimetic OSTM1-T328E/S329D variant and crossed these mice into the *Lrrk1*-null background to evaluate whether restoring OSTM1 phosphorylation could rescue osteoclast function and skeletal homeostasis.

## 2. Materials and Methods

### 2.1. Generation of Lrrk1 Knockout and Ostm1-T328E/S329D Knock-In Mice

**Figure 1:**
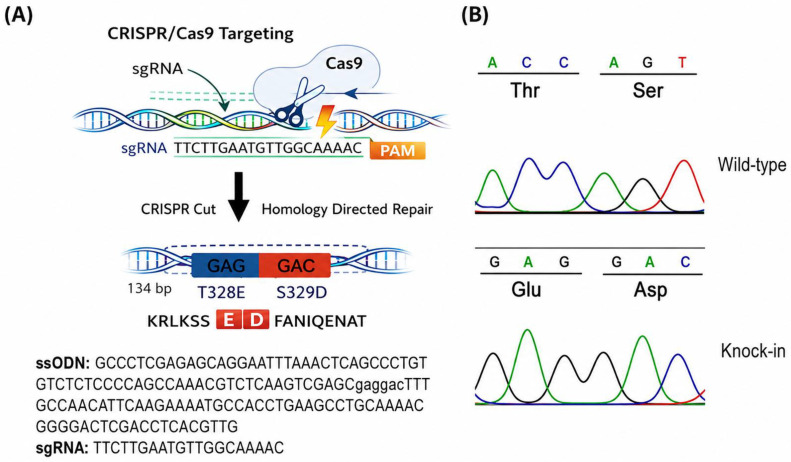
**Generation of *Ostm1*-T328E/S329D knock-in mice by CRISPR/Cas9 technology**. (**A**) The mutations were introduced in exon 6, where the codons for threonine 328 and serine 329 were replaced with GAG GAC for glutamic acid and aspartic acid. A single-guide RNA (sgRNA) was designed to break the double strand DNA surrounding the target residues, and then a single-stranded oligodeoxynucleotide (ssODN) donor template containing the desired nucleotide substitutions was used to introduce the mutations through homology-directed repair. Sequences of ssODN and sgRNA are given at the bottom of the figure. (**B**) Sanger sequencing confirmation of the T328E/S329D knock-in mutations in wild-type and knock-in alleles.

*Lrrk1* KO mice were generated as previously described [5]. *Ostm1-T328E/S329D* KI mice carrying phosphomimetic mutations were produced by CRISPR/Cas9-mediated genome editing at the University of California Irvine Transgenic Core Facility. A single-guide RNA (sgRNA) targeting exon 6 near serine 329 was designed to direct Cas9-induced double-strand break formation (Figure 1A). A 134 nt single-stranded oligodeoxynucleotide (ssODN) donor containing 60 nt homology arms flanking the target site was used to introduce T328E and S329D substitutions via homology-directed repair. The T328E mutation disrupted the upstream PAM sequence, preventing Cas9 re-cleavage of the edited allele. Founder mice were screened using allele-specific PCR (primer sequences in Table 1) and confirmed by Sanger sequencing (Figure 1B). Three independent founder-derived *Ostm1-T328E/S329D* KI mouse lines (Lines A, B, and C) were established and maintained separately. Homozygous *Ostm1* KI mice from each line were used for skeletal phenotypic characterization. Homozygous *Lrrk1*^−/−^; *Ostm1^KI/K^*^I^ (*Lrrk1* KO; *Ostm1* KI) mice were generated through three generations of backcrossing. Age- and sex-matched *Lrrk1* KO and *Lrrk1* KO; *Ostm1* KI littermates were used for phenotypic analyses. Mice were maintained under specific pathogen-free conditions (14 h light/10 h dark cycle, 22 °C) with ad libitum access to standard chow and water. All procedures were approved by the Institutional Animal Care and Use Committee (IACUC) of the VA Loma Linda Healthcare System, Loma Linda, CA, USA (Protocol #1650093/1442).

### 2.2. Plasmids, Cell Lines, Recombinant Proteins, and Antibodies

Lentiviral packaging plasmids (pMD2.G and psPAX2) and pRRLsin-cPPT-SFFV-GFP-wpre were obtained from Addgene (Watertown, MA, USA). The pGEX-4T-1 mRANKL (158–316) plasmid was provided by Dr. Steven Teitelbaum (Washington University School of Medicine) [17]. Coding sequences for mouse LRRK1-FLAG and mutant HA-OSTM1 constructs (T328E/S329D or T328A/S329A) were synthesized by GenScript (Piscataway, NJ, USA) and cloned into the GFP site of pRRLsin-cPPT-SFFV-GFP-wpre vector. HEK293T cells were purchased from ATCC (Manassas, VA, USA). CMG14-10 cells producing recombinant M-CSF were provided by Dr. Sunao Takeshita [18]. FuGene 6 (Promega, Madison, WI, USA) was used for transfections. RANKL-GST fusion protein was expressed in *E. coli* and purified using Next Generation Chromatography (Bio-Rad, Hercules, CA, USA). M-CSF-containing conditioned medium was prepared from CMG14-10 cells as described [18]. Primary antibodies (anti-FLAG M2, anti-HA, anti-β-actin) and secondary antibodies were purchased from Sigma-Aldrich (St. Louis, MO, USA).

### 2.3. Lentivirus Production and Cell Transduction

Lentiviral particles were generated by co-transfecting HEK293T cells with pRRLsin-cPPT-SFFV-GFP-wpre, pRRLsin-cPPT-SFFV-mLRRK1-wpre, pRRLsin-cPPT-SFFV-OSTM1-T328E/S329D-wpre, or pRRLsin-cPPT-SFFV-OSTM1-T328A/S329A-wpre, together with pMD2.G and psPAX2 packaging plasmids, using FuGene 6 (Promega Corporation, Madison, WI, USA) according to manufacturer’s instructions [10]. Viral supernatants were collected 48–72 h post-transfection, filtered through 0.45 µm membranes, and used to infect primary osteoclast precursors as previously described [10].

### 2.4. Cell Culture and Protein Extraction

Primary macrophage precursors were isolated from spleens of 4-week-old WT and *Lrrk1* KO mice and cultured in α-MEM supplemented with 10% FBS, 100 µg/mL streptomycin, 100 U/mL penicillin (Thermo Fisher Scientific, Waltham, MA, USA), 2% CMG14-10 conditioned medium containing ~20 ng/mL M-CSF, and 200 ng/mL RANKL-GST for 6 days [18]. Osteoclast differentiation was assessed by tartrate-resistant acid phosphatase (TRAP) staining; TRAP-positive multinucleated cells (≥3 nuclei) were counted, and cell diameters were measured. Cells were lysed in buffer containing 20 mM Bicine (pH 7.5), 1 mM DTT, 1% protease inhibitor cocktail (Sigma), and 0.6% CHAPS on ice for 20 min. Lysates were clarified by centrifugation at 12,000 rpm for 10 min.

### 2.5. Bone Resorption Pit Assay

Sterilized bone slices were preincubated in αMEM overnight. Osteoclast precursors from the spleens of 4-week-old mice were seeded on bone slices and differentiated for 5–7 days in the presence of M-CSF and RANKL as described above. After maturation, cells were removed and resorption pits visualized by hematoxylin staining. Pit number and total resorbed area were quantified using ImageJ (Version 1.54, NIH). Resorptive pits on bone slices were also imaged using VersaXRM-500 nano-CT (Xradia, Pleasanton, CA, USA) at 0.3 µm voxel resolution.

### 2.6. Western Blot Analysis

Equal amounts of protein (20 µg) were resolved on 4–12% SDS-NuPAGE gels and transferred to 0.45 µm PVDF membranes. Membranes were incubated with primary antibodies (1:5000) against FLAG, HA, or β-actin, followed by HRP-conjugated secondary antibodies (1:10,000). Signals were detected using enhanced chemiluminescence (Thermo Fisher Scientific). β-actin served as a loading control.

### 2.7. Histomorphometric Analysis

Histomorphometry was performed on 12-week-old mice. For dynamic bone formation measurements, mice received intraperitoneal injections of demeclocycline (20 mg/kg), followed by calcein injection (25 mg/kg) 8 days later. Animals were euthanized 2 days after the second injection. Left femurs were fixed in 10% neutral-buffered formalin, dehydrated, and embedded in methyl methacrylate without decalcification. Undecalcified sections were analyzed for fluorochrome labeling in the secondary spongiosa of the distal metaphysis of the femur using OsteoMeasure64 (Version 1.0.4.1, OsteoMetrics, Decatur, GA, USA). Mineral apposition rate (MAR) and bone formation rate per bone surface (BFR/BS) were calculated [19].

For bone resorption parameters, right femurs were decalcified in EDTA, paraffin-embedded, and sectioned at 5 µm. Sections were stained for TRAP, and osteoclast surface per bone surface (Oc.S/BS), erosion surface per bone surface (ES/BS), and eroded surface per osteoclast surface (ES/Oc.S) were quantified in the secondary spongiosa of the distal metaphysis of the femur using OsteoMeasure, following established guidelines [19].

### 2.8. Micro-Computed Tomography (µCT)

Tibias or femurs from 12-week-old mice were fixed in 10% neutral-buffered formalin and stored in PBS prior to scanning. µCT imaging was performed using a Scanco vivaCT45 system (SCANCO Medical AG, Zurich, Switzerland) at 55 kVp and 145 µA [20]. Images were reconstructed and analyzed using Scanco software (Version 1.2.35.0). For trabecular bone analysis of the proximal tibia or distal femur, the region of interest (ROI) began at 0.245 mm for WT mice, and 2.695 mm for osteopetrosis mice with deletion of *Lrrk1* distal to the growth plate and extended 1.225 mm (250 slices; 4.9 µm slice thickness). We set up the different ROIs because the primary spongiosa of the growth plates in *Lrrk1* KO mice extends toward the marrow cavity, and the secondary spongiosa expands near the diaphysis. For comparisons between *Lrrk1* KO and *Lrrk1* KO; *Ostm1-T328E/S329D* KI mice, identical ROI settings (2.695 mm distal to the growth plate and 1.225 mm in length) were used for both groups. Therefore, differences observed between KO and KO/KI mice are not attributable to ROI selection. For analysis of the secondary ossification center (SOC), ROI was defined beginning 30 slices proximal to the growth plate (147 µm total length). Cortical bone parameters were assessed at the femoral mid-diaphysis over 100 slices (1.05 mm). A global threshold of 400 was applied. Quantified parameters included bone mineral density (BMD), bone volume (BV), bone volume fraction (BV/TV), connectivity density (Conn.D), trabecular thickness (Tb.Th), trabecular number (Tb.N), trabecular separation (Tb.Sp), and cortical BV/TV and BMD [6].

### 2.9. Statistical Analysis

Data are presented as mean ± SEM. Statistical significance was determined using student’s *t*-tests or one-way ANOVA with Tukey’s post hoc multiple comparison test using R software (version 4.3.2). A *p*-value < 0.05 was considered statistically significant.

## 3. Results

### 3.1. Expression of OSTM1-T328E/S329D Partially Restores Resorptive Function in LRRK1-Deficient Osteoclasts In Vitro

**Figure 2:**
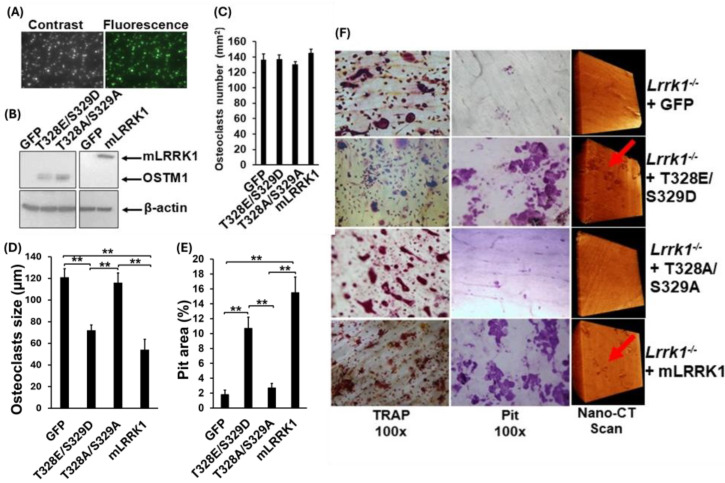
**Overexpression of OSTM1-T328E/S329D partially rescues the resorptive function of LRRK1-deficient osteoclasts in vitro.** Primary osteoclast precursors derived from the spleens of *Lrrk1* knockout (KO) mice were transduced with lentiviruses expressing GFP, mLRRK1, OSTM1-T328E/S329D or OSTM1-T328A/S329A. Transduced cells were differentiated into osteoclasts on bone slices in the presence of M-CSF and RANKL for 6 days, followed by tartrate-resistant acid phosphatase (TRAP) staining and a pit formation assay. (**A**) Representative contrast image of cultured cells and fluorescent image of lentivirus infected GFP-positive cells. (**B**) Expression of mLRRK1 and OSTM1 mutant proteins detected by monoclonal anti-flag and polyclonal anti-HA antibodies, respectively, by two separated Western blotting membranes, with their own β-actin loading controls. To avoid signal saturation, 50% less protein was loaded for β-actin. (**C**–**E**) Quantification of osteoclast number, osteoclast size and pit area, respectively. (**F**) Representative images of TRAP-positive multinucleated osteoclasts and resorptive pits stained by hematoxylin and scanned by nano-computed tomography (nano-CT). Data are presented as mean ± SEM (*n* = 6). Statistical significance was determined by one-way ANOVA followed by Tukey’s multiple comparison test. ** indicates *p* < 0.01.

To determine whether impaired OSTM1 signaling contributes to the defective resorptive activity of osteoclasts lacking LRRK1, we performed rescue experiments using lentivirus-mediated overexpression of a phosphomimetic OSTM1 variant (T328E/S329D) or a dephosphomimetic variant (T328A/S329A). LRRK1-deficient precursors expressing mouse *Lrrk1* (*mLrrk1*) served as a positive control. Lentiviral transduction efficiency was high, as indicated by robust GFP expression in nearly all infected precursors (Figure 2A). Western blot analyses confirmed that the expression level of OSTM1-T328A/S329A was slightly higher than OSTM1-T328E/S329D (Figure 2B and Figure S3).

Although mature osteoclast numbers were similar across groups, expression of OSTM1-T328E/S329D—but not OSTM1-T328A/S329A—restored resorptive activity in LRRK1-deficient osteoclasts (Figure 2C–F). Resorption pit area increased approximately seven-fold in OSTM1-T328E/S329D-expressing cells relative to GFP controls. As expected, mLRRK1 expression rescued resorption to a similar extent, increasing pit area by approximately eight-fold (Figure 2F). Nano-CT imaging further confirmed that OSTM1-T328E/S329D-expressing osteoclasts generated larger and deeper pits than cells expressing GFP or OSTM1-T328A/S329A. In addition, osteoclasts expressing either mLRRK1 or OSTM1-T328E/S329D were smaller (65–75 µm), whereas GFP- or OSTM1-T328A/S329A-expressing osteoclasts were enlarged (~120 µm), consistent with impaired osteoclast function.

### 3.2. Ostm1-T328E/S329D Knock-In Mice Exhibit Normal Skeletal Development and Bone Remodeling

To determine whether phosphomimetic OSTM1 expression affects skeletal development or homeostasis, we generated three independent *Ostm1-T328E/S329D* KI mouse lines using CRISPR/Cas9. Heterozygous intercrosses produced WT, heterozygous, and homozygous KI offspring at expected Mendelian ratios. All KI mice were viable, fertile, and phenotypically normal at 11 weeks of age. Sequencing full-length *Ostm1* cDNA confirmed successful introduction of the T328E and S329D substitutions in all lines.

**Figure 3:**
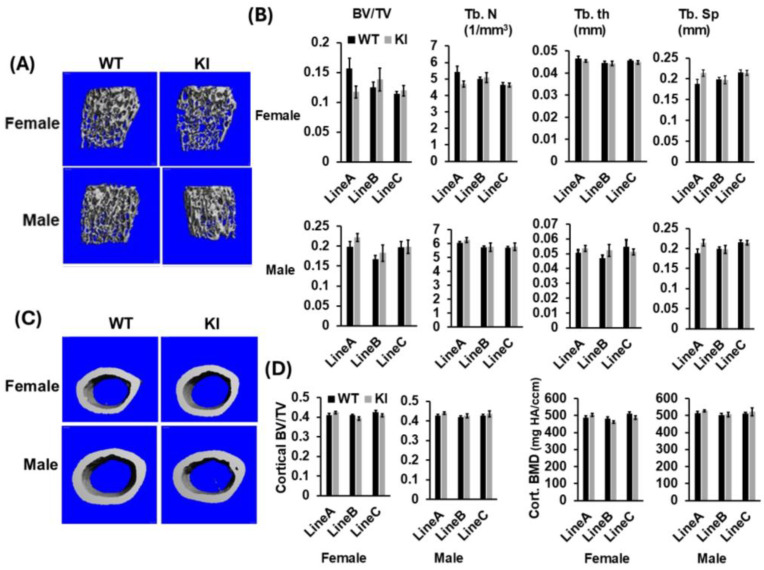
**There are no significant changes in trabecular and cortical bone parameters in the distal femur of *Ostm1-T328E/S329D* knock-in mice.** Bone density of the femur from 12-week-old homozygous *Ostm1*-T328E/S329D knock-in (KI) mice and wild-type (WT) littermates was measured by micro-computed tomography (µCT) scanning. (**A**) Longitudinal sections of the trabecular bone at the distal femurs from the WT and homozygous *Ostm1*-*T328E/S329D* KI females and males from line A, one of three independent Ostm1-T328E/S329D knock-in mouse lines generated by CRISPR/Cas9. (**B**) Quantitative data of trabecular bone volume/tissue volume (BV/TV), trabecular number (Tb.N), trabecular thickness (Tb.Th), and trabecular separation (Tb.Sp). (**C**) Cross sections of cortical bone of the midshaft femurs of WT and homozygous *Ostm1*-*T328E/S329D* KI females and males from line A. (**D**) Cortical BV/TV and bone mineral density (BMD) of the femurs of the WT and homozygous *Ostm1*-*T328E/S329D* KI females and males. Data are presented as mean ± SEM (*n* = 7–10).

Both male and female KI mice displayed normal body weight, body length, and femur length compared with WT littermates (Supplemental Figure S1A–C), with no line-dependent variation. Micro-CT analysis of the distal femur revealed no significant differences in trabecular bone parameters between KI and WT mice of either sex. In female line A mice, BV/TV was reduced by 17%, accompanied by a 10% decrease in Tb.N and a 10% increase in Tb.Sp; however, none of these changes reached statistical significance (Figure 3A,B). Trabecular thickness (Tb.Th) was unchanged. No trabecular abnormalities were detected in males. Cortical BV/TV and BMD were comparable between KI and WT mice in both sexes (Figure 3C,D).

**Figure 4:**
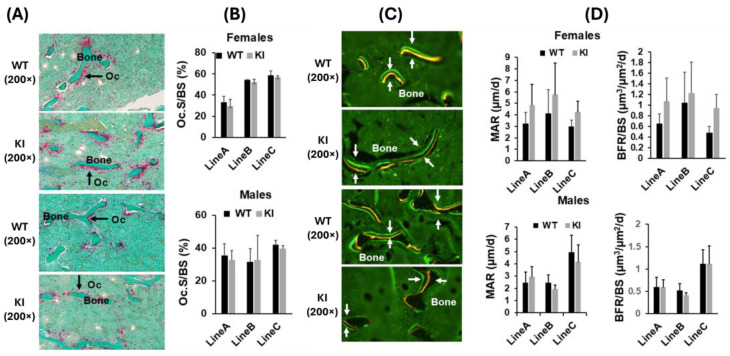
**There are no significant changes in bone formation parameters in both male and female *Ostm1-T328E/S329D* knock-in mice at age of 12 weeks.** Homozygous *Ostm1*- *T328E/S329D* KI and WT gender-matched mice were double dye-labeled with demeclocycline and calcein. The mice were euthanized 2 days after the second injection for histomorphometry analyses. (**A**,**B**) Representative images of TRAP-positive osteoclasts and quantitative data of the osteoclast surface/bone surface (Oc.S/BS) in the femurs of the WT and homozygous *Ostm1*-*T328E/S329D* KI female and male mice. Arrows indicate the TRAP-positive osteoclast (Oc) on bone surface. (**C**,**D**) Representative images of double labeling and quantitative data of the mineral apposition rate (MAR) and the bone formation rate/bone surface (BFR/BS) in the femur of WT and homozygous *Ostm1*-*T328E/S329D* KI female and male mice. Data are presented as mean ± SEM (*n* = 6–10 mice). Arrows indicate newly formed bones between two color labels.

Histomorphometric analyses further confirmed normal skeletal homeostasis in KI mice. TRAP staining showed normal osteoclast differentiation and bone surface association in both sexes (Figure 4A). The Oc.S/BS was unchanged relative to WT controls (Figure 4B). Dynamic histomorphometry revealed no significant differences in MAR or BFR/BS between KI and WT mice at 12 weeks of age (Figure 4C,D).

### 3.3. Expression of OSTM1-T328E/S329D in Lrrk1 KO Mice Increases Trabecular Separation and Reduces Connectivity

**Figure 5:**
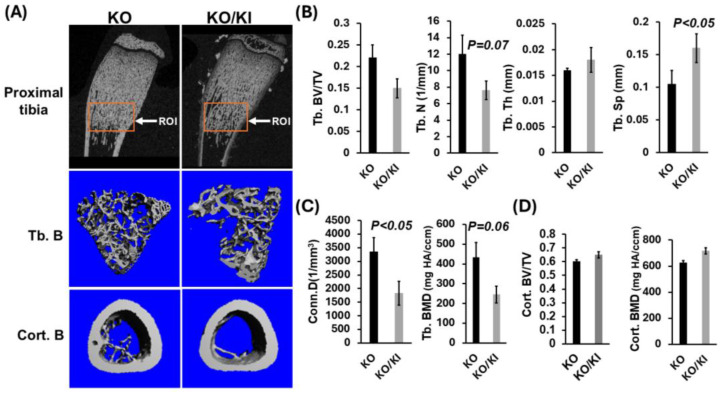
**Expression of OSTM1-T328E/S329D variant in *Lrrk1* KO female mice elevates trabecular separation and reduces connectivity density.** Bone phenotypes of the proximal tibias from 12-week-old female *Lrrk1* KO females and gender-matched *Lrrk1* KO; *Ostm1*-T328E/S329D KI (KO/KI) littermates were analyzed by µCT. (**A**) Representative images of the longitudinal sections of the proximal tibias, cross-sections of the tibial secondary spongiosa, and the midshaft cortical bone, respectively. Arrows indicate the regions of interest (ROI) for trabecular measurements. (**B**,**C**) Quantitative measurements of trabecular BV/TV, Tb.N, Tb.Th, Tb.Sp, connectivity density (Conn.D), and bone mineral density (BMD). (**D**) Cortical BV/TV and BMD. Data are presented as mean ± SEM (*n* = 9). *p* < 0.05 indicates statistically significant as compared to KO mice.

To evaluate whether phosphomimetic OSTM1 expression modifies the osteopetrotic phenotype of *Lrrk1*-deficient mice, we generated homozygous *Lrrk1* KO; *Ostm1-T328E/S329D* KI (KO/KI) mice and *Lrrk1* KO littermates (Supplemental Figure S2). Because no line-dependent skeletal differences were observed, line A was used for all subsequent analyses. Micro-CT analysis of the proximal tibias from 12-week-old females revealed the expected osteopetrotic phenotype in both KO and KO/KI mice, with dense trabecular bone occupying the marrow cavity (Figure 5A). Quantitative analysis showed that KO/KI mice exhibited significantly increased trabecular separation (Tb.Sp; +53%) and reduced connectivity density (Conn.D; –45%) compared with KO mice (Figure 5B). Tb.N and BMD were also reduced by 37% and 44%, respectively, whereas BV/TV and Tb.Th remained unchanged (Figure 5B,C). Cortical BV/TV and BMD were similar between genotypes (Figure 5D). Analysis of the SOC region of the tibia revealed no significant differences in BMD or trabecular architecture between KO and KO/KI mice. Trabecular BV/TV, Tb.N, Tb.Th, Tb.Sp, and Conn.D were comparable between the two genotypes (Supplemental Figure S4). These findings indicate that the expression of the OSTM1-T328E/S329D variant does not significantly alter the SOC formation and remodeling in *Lrrk1*-deficient mice.

### 3.4. Expression of OSTM1-T328E/S329D Enhances Osteoclast Activity and Bone Formation in Lrrk1 KO Mice

**Figure 6:**
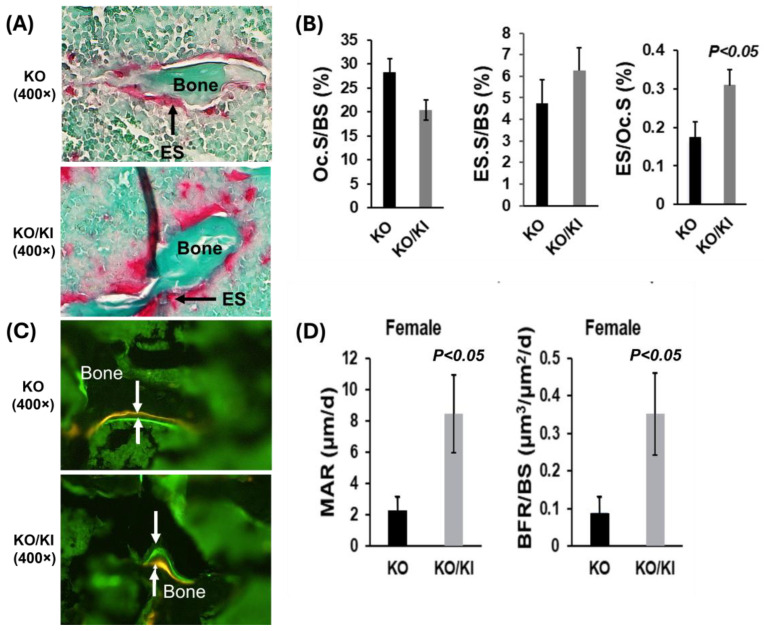
**Expression of OSTM1-T328E/S329D variant in *Lrrk1* KO female mice increases osteoclast activity.** Bone resorption and bone formation parameters were analyzed by histomorphometry analysis on tissue sections of the femur from 12-week-old *Lrrk1* KO and *Lrrk1* KO; *Ostm1*-T328E/S329D KI female mice. (**A**) Representative TRAP-stained sections. Arrows indicate TRAP-positive osteoclast and erosion surface (ES). (**B**) Quantification of Oc.S/BS, erosion surface per bone surface (ES/BS), and eroded surface per osteoclast surface (ES/Oc.S). (**C**) Representative images of demeclocycline/calcein double labeling. Arrows indicate newly formed bones. (**D**) Quantification of MAR and bone BFR/BS. Data are presented as mean ± SEM (*n* = 9). *p* < 0.05 indicates statistically significant as compared to KO mice.

To determine whether altered trabecular architecture in KO/KI mice was associated with changes in osteoclast activity, we performed histomorphometric analyses on femoral sections from 12-week-old females. TRAP staining revealed osteoclasts along trabecular surfaces in both KO and KO/KI mice (Figure 6A). The Oc.S/BS was slightly reduced in KO/KI mice but did not reach statistical significance (Figure 6B). However, TRAP staining intensity appeared stronger in KO/KI osteoclasts, suggesting enhanced enzymatic activity. Consistent with this observation, erosion surface per bone surface (ES/BS) was increased by ~20%, and the proportion of active osteoclasts (ES/Oc.S) was increased by ~40% in KO/KI mice relative to KO mice (Figure 6B). Dynamic histomorphometry further demonstrated significantly elevated MAR and BFR/BS in KO/KI mice, indicating increased bone formation (Figure 6C,D).

## 4. Discussion

In this study, we identified phosphorylation of OSTM1 at threonine 328 and serine 329 as a previously unrecognized regulatory mechanism required for optimal osteoclast resorptive function. Using both in vitro and in vivo models of OSTM1 KI gain-of-function, we have demonstrated that phosphomimetic OSTM1 (T328E/S329D) partially restored bone resorption in *Lrrk1*-deficient osteoclasts, supporting the conclusion that OSTM1 is an important downstream effector of the LRRK1 signaling pathway. These findings together with our previous observation provide mechanistic insight into how LRRK1 regulates lysosomal trafficking, positioning and secretion—processes essential for osteoclast-mediated bone resorption [10].

Previous work established that intrinsic OSTM1 deficiency is sufficient to cause severe osteopetrosis in mice and humans. A KI mouse model mimicking a human OSTM1 transmembrane-domain mutation (exon 5) recapitulated the osteopetrotic phenotype, with mature osteoclasts exhibiting impaired lysosomal distribution and defective exocytosis [15,16]. Motivated by these observations and our discovery that OSTM1 phosphorylation is severely compromised in RANKL-activated LRRK1-deficient osteoclasts, we focused on defining the role of OSTM1 as a mediator of LRRK1 function. To this end, we engineered a phosphomimetic OSTM1 KI mouse model and evaluated whether restoring OSTM1 phosphorylation could rescue osteoclast defects caused by LRRK1 loss.

Our in vitro rescue experiments revealed that overexpression of OSTM1-T328E/S329D, but not the dephosphomimetic OSTM1-T328A/S329A, significantly enhanced pit formation in *Lrrk1*-deficient osteoclasts. Although the degree of rescue did not fully reach that of WT osteoclasts, the substantial increase in pit number and depth indicates that phosphorylation of OSTM1 at these residues is functionally important. The partial nature of the rescue suggests that LRRK1 regulates additional substrates or pathways required for full osteoclast activity. This is consistent with prior work showing that LRRK1 signaling targets other regulatory proteins critical for cytoskeletal dynamics, endosome sorting, lysosomal positioning, and ruffled border formation through post-translational modification [21].

Prior to crossing *Ostm1-T328E/S329D* KI mice with *Lrrk1* KO mice for skeletal phenotype evaluation, we first determined if KI of mutant *Ostm1-T328E/S329D* gene influences normal skeletal development and bone remodeling. Our studies using three independent lines of mice with *Ostm1-T328E/S329D* revealed no effect on cortical or trabecular bone phenotypes in adult mice. Accordingly, neither bone formation nor resorption parameters were affected, indicating that phosphomimetic OSTM1 does not perturb physiological osteoclast function in the presence of endogenous *Lrrk1*. However, when introduced into the *Lrrk1*-deficient background, OSTM1-T328E/S329D expression altered trabecular architecture by increasing trabecular separation and reducing connectivity density. Histomorphometric analyses revealed elevated erosion surface and increased osteoclast activity, accompanied by enhanced bone formation. These findings suggest that phosphomimetic OSTM1 partially restores aspects of osteoclast function in vivo, but the rescue is incomplete and may lead to compensatory increases in bone formation.

Studies on genetic loci that contribute to variation in peak BMD differences using mouse strains as well as human populations have revealed that genetic regulation of skeletal development varies depending on the skeletal site. Thus, it is not surprising that the magnitude of BMD increase varies depending on the skeletal site in *Lrrk1* KO mice. Our findings also highlight a fundamental regional divergence in how skeletal tissues respond to modulation of OSTM1 function. Although expressing phosphomimic OSTM1 normalizes osteoclast function, this rescue does not translate into progression of the SOC. This lack of responsiveness likely reflects the distinct cellular and molecular dependencies underlying SOC development. SOC formation is driven primarily by chondrocyte hypertrophy, matrix remodeling, and subsequent osteoblast-mediated bone deposition—processes that occur largely independent of osteoclast-specific regulatory pathways. [22,23] Because OSTM1 acts predominantly within osteoclasts, restoring its phosphorylation corrects defects restricted to bone-resorbing cells but does not influence the chondrocyte to osteoblast cascades that are essential for SOC initiation and expansion. Consequently, regions that rely heavily on chondrocyte- and osteoblast-dependent ossification remain unaffected by OSTM1 rescue, underscoring a clear mechanistic separation between osteoclast-mediated pathways and the developmental programs governing epiphyseal ossification.

In terms of the mechanism by which LRRK1-induced phosphorylation of OSTM1 can influence its activity, it is well established that phosphorylation can modulate the nature and the strength of protein–protein interactions by either affecting the binding energy of the complex or changing the protein conformation, thereby regulating protein binding, stability and function [24]. Accordingly, we predict that serine/threonine phosphorylation of OSTM1 by LRRK1 may stabilize OSTM1 and promote its interaction with the CLC-7 subunit, thereby regulating transport and secretion of acidic lysosomes [25]. Consistent with an essential role of the CLC-7/OSTM1 complex in osteoclast biology are the findings that OSTM1 complex is required to stabilize CLC-7 complex [26]. Loss-of-function mutations in either component cause severe osteopetrosis in humans and mice, and our data now indicate that phosphorylation of OSTM1 may contribute to the stability and function of this complex [11]. The identification of OSTM1 phosphorylation as a regulatory mechanism opens new avenues for understanding how lysosomal acidification and trafficking are coordinated during bone resorption.

Our finding that *Lrrk1* KO mice expressing mutant OSTM1-T328E/S329D exhibit both increased osteoclast activity and bone formation is particularly intriguing. It is known that osteoblast-mediated bone formation is tightly coupled to osteoclast-mediated bone resorption and that coupling factors released from active osteoclasts or from bone matrix during bone resorption contribute to this [27,28]. Thus, even partial restoration of resorptive function may be sufficient to stimulate downstream anabolic responses. This aligns with the preserved osteoclast-coupled bone formation observed in *Lrrk1* KO mice treated with anabolic PTH, further supporting the idea that LRRK1 regulates osteoclast function without impairing osteoblast responsiveness [5].

In this study, although phosphomimetic OSTM1 markedly improved resorptive activity in LRRK1-deficient osteoclasts in vitro, the in vivo rescue of defective bone resorption in *Lrrk1* KO; *Ostm1-T328E/S329D* KI mice was modest at best. There are several potential explanations for why expression of mutant OSTM1 did not rescue skeletal phenotype in *Lrrk1* KO mice that include: first, phosphomimetic substitutions of OSTM1 employed in this study only approximate the negative charge of phosphorylation and may not fully reproduce the conformational or dynamic changes induced by true kinase-mediated OSTM1 modification. Thus, OSTM1-T328E/S329D may only partially mimic the native phosphorylated state in vivo. Second, LRRK1 regulates multiple components of osteoclast cytoskeletal and lysosomal machinery. While OSTM1 is a key downstream effector, it is unlikely to be the sole mediator of LRRK1 function. Additional LRRK1 substrates—such as proteins involved in actin ring assembly, endosome sorting, vesicle trafficking, or ruffled border formation—may be required for full restoration of osteoclast activity [21]. Third, the in vitro environment isolates osteoclast-intrinsic mechanisms, whereas the in vivo setting integrates systemic cues, microenvironmental constraints, osteoclast-chondrocyte-endothelial cell communications, and osteoclast–osteoblast coupling. Even partial restoration of resorption may trigger compensatory increases in bone formation, as observed in the *OSTM1-T328E/S329D* KI mice, thereby masking or counterbalancing structural improvements in trabecular bone. Fourth, in the in vitro experiments, osteoclast precursors were transduced with high-titer lentivirus, resulting in supraphysiological expression of *OSTM1-T328E/S329D*. This level of expression can amplify partial functional effects and compensate for upstream defects. In contrast, the KI mice have only two endogenous alleles encoding *Ostm1-T328E/S329D* expression under the native promoter, which may be insufficient to fully overcome the severe osteoclast defects caused by complete LRRK1 loss. Finally, the severe osteopetrotic phenotype of *Lrrk1* KO mice may limit the extent to which rescue of any single downstream effector can fully reverse architectural abnormalities caused by lack of LRRK1 function, thus underscoring the complexity of the LRRK1 signaling network and the need to identify the additional regulatory nodes involved in LRRK1 action in bone.

## 5. Conclusions

In summary, this work establishes OSTM1 phosphorylation at T328/S329 as an important component of the LRRK1 signaling axis in osteoclasts. By demonstrating that phosphomimetic OSTM1 partially restores osteoclast function in LRRK1-deficient cells and mice, we provide mechanistic insight into the molecular basis of LRRK1-mediated bone resorption. These findings advance our understanding of osteoclast biology and highlight the LRRK1–OSTM1 pathway as a promising target for therapeutic modulation in metabolic bone diseases characterized by impaired or excessive bone resorption. Because expression of phosphomimetic OSTM1 alone is insufficient to fully restore skeletal phenotype in *Lrrk1* KO mice, future studies aimed at identifying additional downstream components of the LRRK1 signaling network will be essential for developing targeted therapies for osteopetrosis and other disorders of bone remodeling.

## Figures and Tables

**Table 1:** Primers used for genotyping.

Primer	Sequence	Allele	PCR Product
Forward	5′-AGGAAAATTGGATCAAGCCATGT-3′	Wild-type	301 bp
Reverse	5′-ATAGGTCTGCAGTCCCAACATT-3′	Common	
Forward	5′-GTCTCAAGTCGAGCGAGGAC-3′	Mutant	177 bp

## Data Availability

The datasets generated in this study are available from the corresponding author (W.X.; weirong.xing@va.gov) upon request.

## Supplementary Materials

The following supporting information can be downloaded at: https://www.mdpi.com/article/10.3390/biology15120964/s1, Supplemental Figure S1: There are no significant changes in body weight, body length and femur length in both male and female *Ostm1*-*T328E/S329D* knock-in mice at age of 12 weeks; Supplemental Figure S2: Breeding strategy of generation of *Lrrk1* KO; *Ostm1*-*T328E/S329D KI* mice; Supplemental Figure S3: Overexpression levels of OSTM1 proteins in osteoclasts; Supplemental Figure S4: There are no significant changes in BMD and trabecular architectures in the secondary ossification center (SOC) of the proximal tibia in female *Ostm1*-*T328E/S329D* knock-in mice.
