# Supplementary material for: Expression of Phosphomimetic OSTM1-T328E/S329D Variant Partially Restores Bone Resorption Defect in LRRK1-Deficient Mice
Source: Biology (Basel). 2026 Jun 19;15(12):964. doi: 10.3390/biology15120964 (PMC13296195; doi:10.3390/biology15120964)
Supplement: Supplementary file 1 [file biology-15-00964-s001.zip › biology-4342073-supplementary.pdf]

Supplemental Figure S1

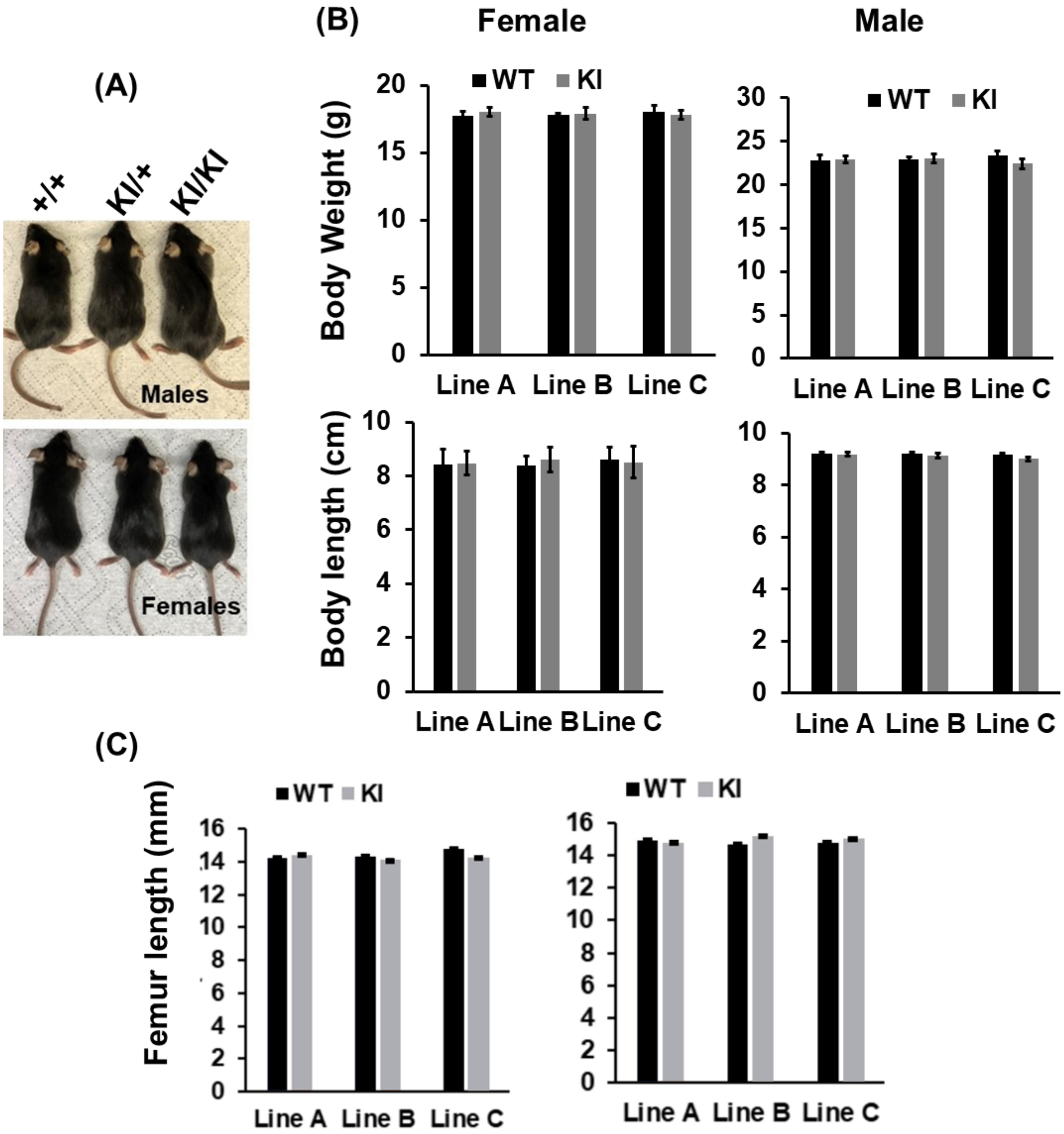

Supplemental Figure S1. There are no significant changes in body weight, body length and femur length in both male and female *Ostm1-T328E/S329D* knock-in mice at age of 12 weeks. Body weight, body length, and femur length were assessed in three independent *Ostm1-T328E/S329D* KI lines and compared with WT gender-matched littermates at 10 weeks of age. (A, B) Body weight in females and males. [C, D] Body length in females and males. (E, F) Femur length in females and males. Data are presented as mean ± SEM (*n* = 9–17).

Supplemental Figure S2

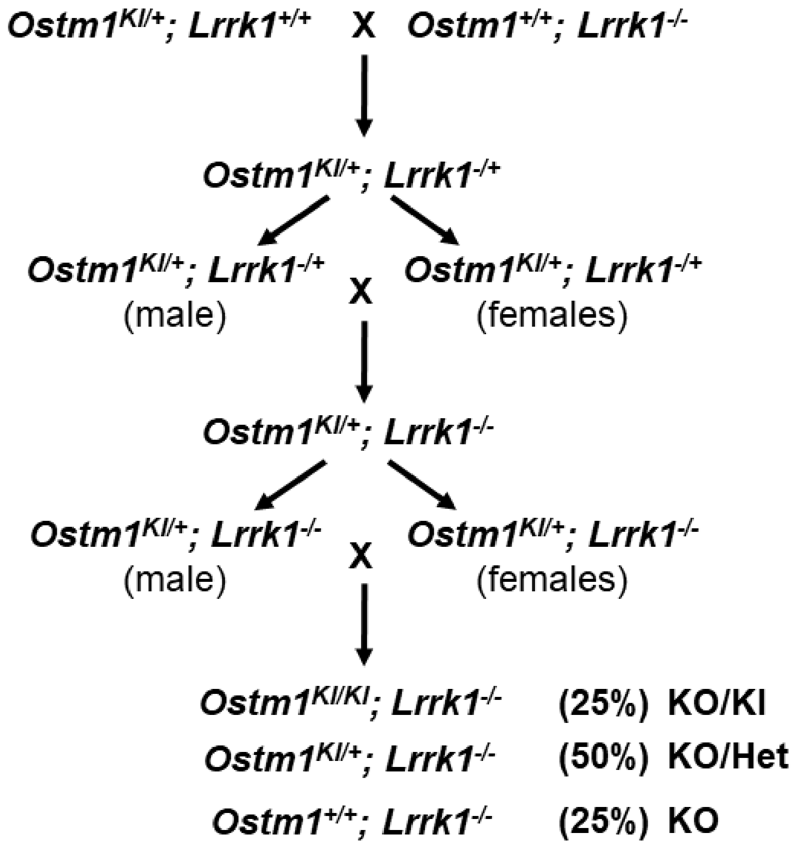

Supplemental Figure S2. Breeding strategy of generation of *Lrrk1* KO; *Ostm1-T328E/S329D* KI mice. Homozygous *Lrrk1* KO; *Ostm1*-T328E/S329D KI (KO/KI) mice and *Lrrk1* KO control littermates were generated by 3 generations of breeding. *Ostm1*$^{+/+}$: WT alleles of *Ostm1*. *Ostm1*$^{KI/+}$: Heterozygous alleles of *Ostm1*-T328E/S329D. *Ostm1*$^{KI/KI}$: Homozygous alleles of *Ostm1*-T328E/S329D. *Lrrk1*$^{+/+}$: WT alleles of *Lrrk1*. Lrrk1$^{-/+}$: Heterozygous alleles of *Lrrk1* deletion. Lrrk1$^{-/-}$: Homozygous alleles of *Lrrk1* deletion.

Supplementary Figure S3

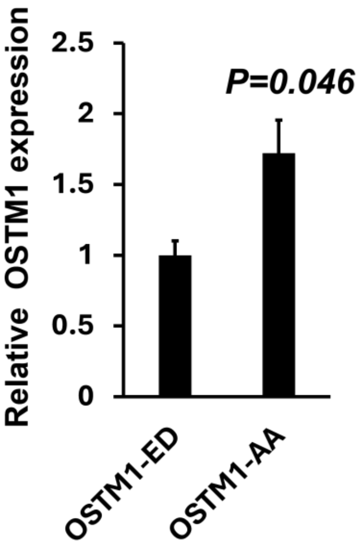

Supplemental Figure S3: Overexpression levels of OSTM1 proteins in osteoclasts. OSTM1 expression were detected by Western blots and the protein levels were normalized to β-actin. Data are presented as mean ± SEM (n = 3). P value was determined using a *Student's t*-test.

Supplemental Figure S4

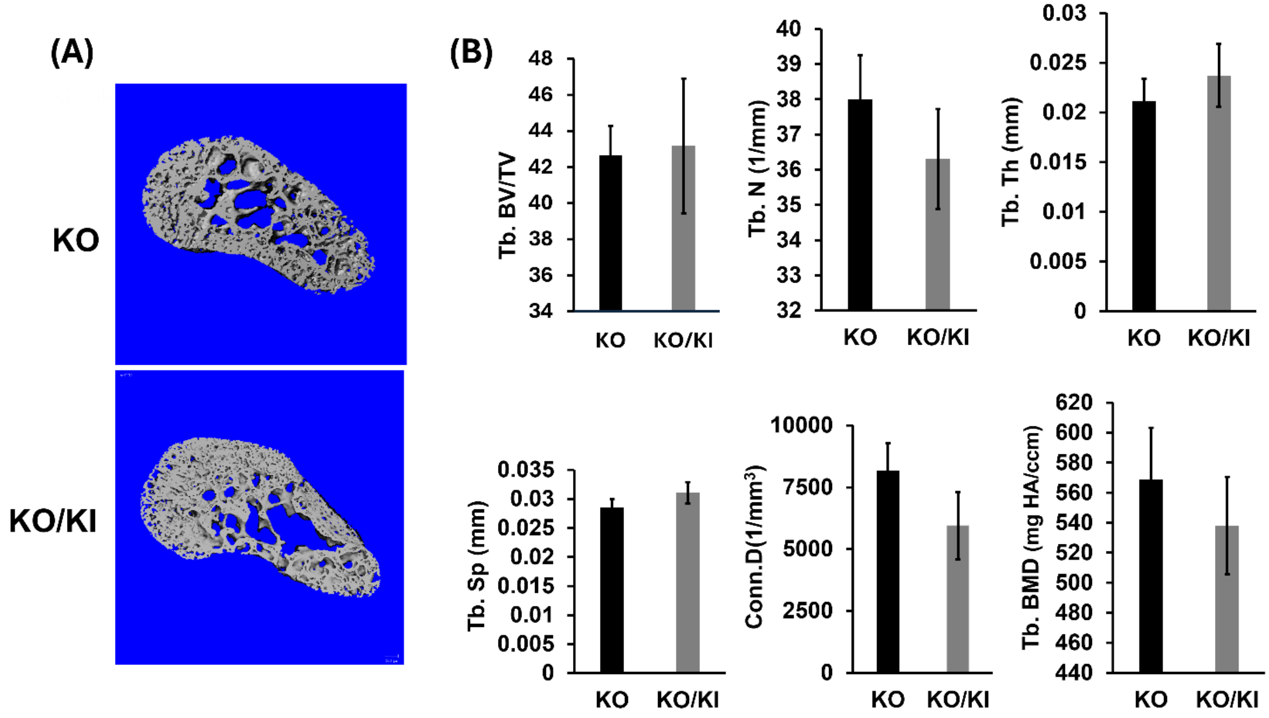

Supplemental Figure S4: There are no significant changes in BMD and trabecular architectures in the secondary ossification center (SOC) of the proximal tibia in female Ostm1-T328E/S329D KI mice. (A) Representative cross μCT images of the SOCs from 12-week-old female KO and KO/KI mice. (B) Quantitative measurements of BV/TV, Tb.N, Tb.Th, Tb.Sp, Conn.D, and BMD are shown. Data are presented as mean ± SEM (n = 9).

Original Western blot images

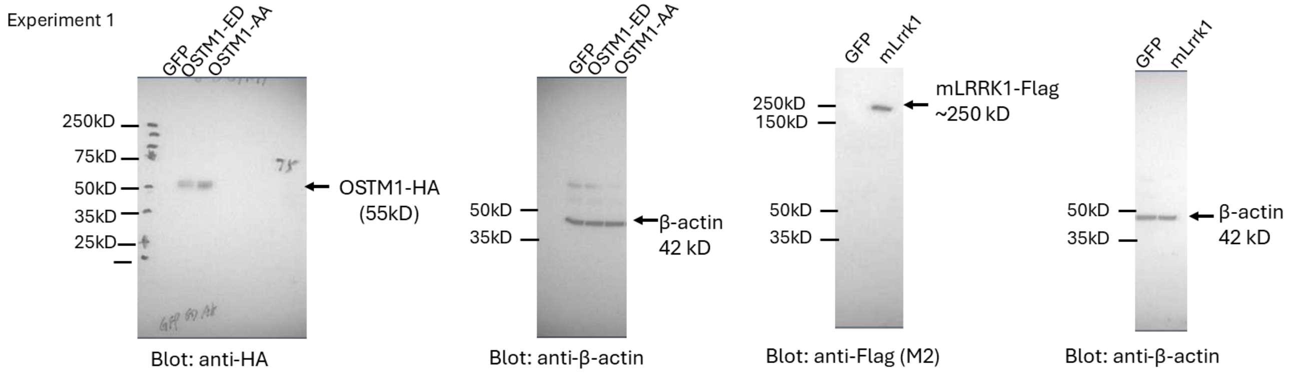

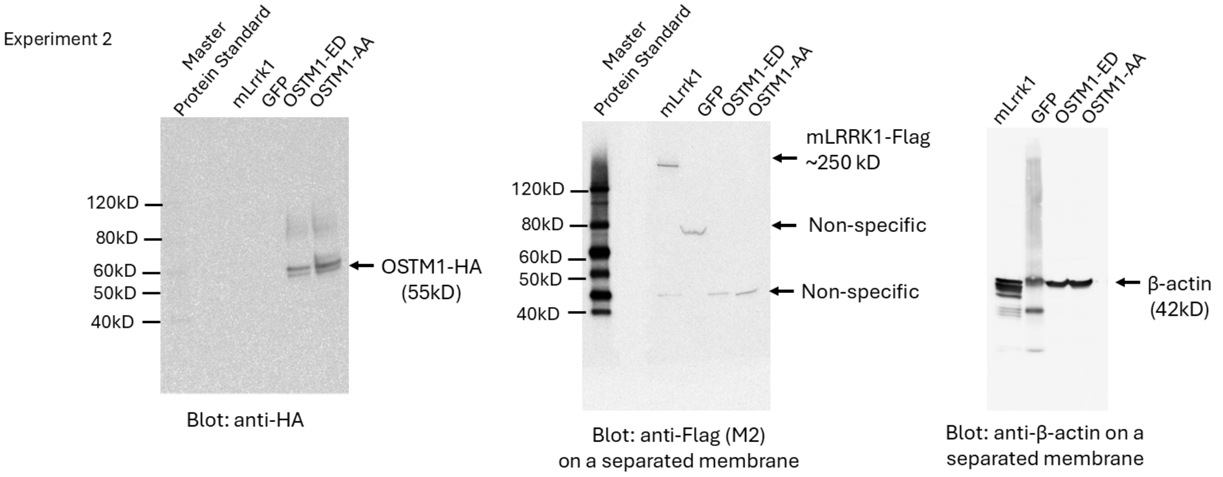

Experiment 3

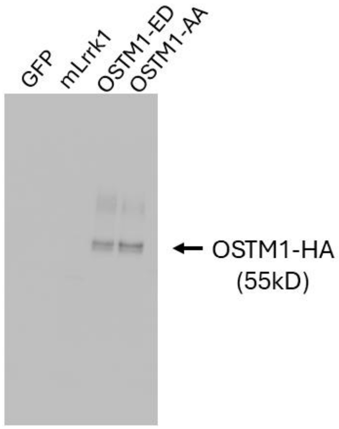

Blot: anti-HA
(rabbit polyclonal antibody)

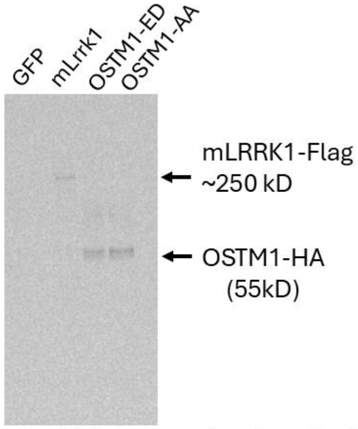

Blot: anti-Flag monoclonal antibody
after blotting with anti-HA on the same membrane

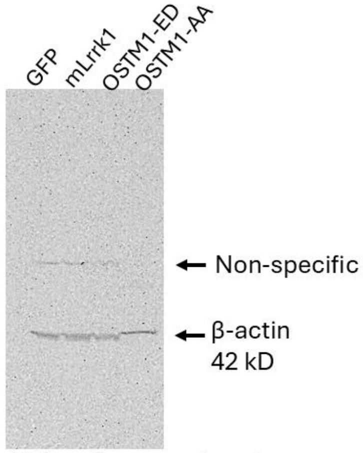

Blot: anti-β-actin monoclonal
antibody on a separated membrane,
with 50% less loading
